# Circuit training enhances function in patients undergoing total knee arthroplasty: a retrospective cohort study

**DOI:** 10.1186/s13018-017-0654-4

**Published:** 2017-10-19

**Authors:** Wei-Hsiu Hsu, Wei-Bin Hsu, Wun-Jer Shen, Zin-Rong Lin, Shr-Hsin Chang, Robert Wen-Wei Hsu

**Affiliations:** 1 Sports Medicine Center, Chang Gung Memorial Hospital, No. 6, West Section, Chia-Pu Road, Putz City, 61363 Chiayi Country Taiwan, Republic of China; 2 Department of Orthopaedic Surgery, Chang Gung Memorial Hospital, No. 6, West Section, Chia-Pu Road, Putz City, 61363 Chiayi Country Taiwan, Republic of China; 3 PO CHENG Orthopedic Institute, No. 100, Bo-ai 2nd Road, Kaohsiung, 81357 Zuoying District Taiwan, Republic of China; 4 Department of Athletic Sports, National Chung Cheng University, No.168, University Road, Minhsiung Township, 62102 Chiayi Country Taiwan, Republic of China

**Keywords:** Circuit training, Total knee arthroplasty, Motion analysis, KOOS, SF-36

## Abstract

**Background:**

The number of patients receiving total knee arthroplasty (TKA) has been rising every year due to the aging population and the obesity epidemic. Post-operative rehabilitation is important for the outcome of TKA.

**Methods:**

A series of 34 patients who underwent primary unilateral TKA was retrospectively collected and divided into either exercise group (*n* = 16) and control group (*n* = 18). The exercise group underwent a 24-week course of circuit training beginning 3 months after total knee arthroplasty (TKA). The effect of circuit training on TKA patients in terms of motion analysis, muscle strength testing, Knee injury and Osteoarthritis Outcomes Score (KOOS) questionnaire and patient-reported outcome measurement Short-Form Health Survey (SF-36) at the pre-operation, pre-exercise, mid-exercise, and post-exercise.

**Results:**

Motion analysis revealed the stride length, step velocity, and excursion of active knee range of motion significantly improved in the exercise group when compared to those in the control group. KOOS questionnaire showed a greater improvement in pain, ADL, and total scores in the exercise group. The SF-36 questionnaire revealed a significant improvement in general health, bodily pain, social function, and physical components score in the exercise group.

**Conclusions:**

The post-operative circuit training intervention can facilitate recovery of knee function and decrease the degree of pain in the TKA and might be considered a useful adjunct rehabilitative modality. The ultimate influence of circuit training on TKA needs further a prospective randomized clinical trial study and long-term investigation.

**Trial registration:**

NCT02928562

## Background

Total knee arthroplasty (TKA) is a well-accepted procedure for the treatment of advanced osteoarthritis of the knee joint [1], with good long-term survivorship of the implants and satisfactory surgical outcome [2]. The number of patients receiving TKA has been rising every year due to the aging population and the obesity epidemic [3–5]. Enhancing the recovery process after TKAs is an important issue. Physical exercise was suggested to enhance muscular strength, muscular endurance, cardiovascular fitness, flexibility, agility, balance, and coordination in the healthy aged population [6]. Physical exercise could further increase the range of motion and quadriceps strength in TKA patients [7]. However, these effects of post-operative exercise were usually limited by a short hospital stay, impaired exercise adherence, and in compliance to exercise regimens [8–10].

A more balanced exercise approach is therefore recommended for TKA patients [11]. Circuit training comprised of different exercise principles, including stretching exercise, aerobic training, and resistant training. Multiple stations for training different muscle groups, stretching exercise, and aerobic exercise was employed to progress towards cardiovascular fitness and muscle strength [12]. We had previously reported its positive effect on body composition improvement in overweight women [9]. Meanwhile, good exercise adherences were also observed with concomitant increases in the mental domain of functional score, since the incidence of female osteoarthritis (OA) is twice that of men [13] and the number undergoing TKA in the female is about twice in men between 2001 and 2010 [14]. The retrospective study focused on the female and assessed the feasibility and effect of post-operative circuit training intervention on TKA. The circuit training intervention would carry out at 3 months after the operation for 6 months. The assessments of this intervention were measured using motion analysis and muscle strength testing, KOOS questionnaire, and patient-reported outcome measurement Short-Form Health Survey (SF-36). The goal was to demonstrate the effect, if any, that circuit training intervention might have on post-operative knee functional recovery and daily activities.

## Methods

### Participants

The aim was to demonstrate the effect, if any, that circuit training intervention might have on post-operative knee functional recovery and daily activities in female TKA.

The inclusion criteria of the study included end-stage OA and female. Patients with diabetes, neuromusculoskeletal disorders, severe chronic medical disease, history of fracture of a lower limb, artificial limb, and being otherwise unsuitable for exercise training were excluded.

From October 2013 to August 2015, a consecutive cohort of 34 patients underwent TKA (16 in the exercise group and 18 in the control group) at Chang Gung Memorial Hospital Chiayi branch was included in the current study.

The participants in the exercise group practiced a 24-week circuit training intervention while the control group followed the routine post-operative rehabilitation protocol, including quadriceps training and range of motion exercise during the same time period.

### Intervention

The circuit training program included stretching, aerobic training, and resistance training. The order of practice was performed in the following pattern: stretching/aerobic training/resistance training/aerobic training/resistance training/stretching. Each exercise was performed for 10 min, with a 30 s rest period between each exercise, and the program was carried out three times a week for 24 weeks. Aerobic training consisted of riding an exercise bike with the intensity of 60–80% target heart rate, and the resistance training was performed on hydraulic resistance equipment (AGOSS, Taipei, Taiwan) set at an intensity of 60–80% one repetition maximum. Six exercise machines were randomly chosen from ten exercise machines which included six types of equipment for the upper limbs and four for the lower limbs. The program was hospital-based and supervised by one exercise therapist at the Sports Medicine Center of Chang Gung Memorial Hospital Chiayi branch.

### Objectives

The aim of the study was to assess the feasibility and effect of post-operative circuit training intervention on TKA women. The effect of this intervention was measured using motion analysis, muscle strength testing, Knee injury and Osteoarthritis Outcome Score (KOOS) questionnaire and Short-Form Health Survey (SF-36) measurement patient-reported outcome. The goal was to demonstrate the effect, if any, that circuit training intervention might have on post-operative knee functional recovery and daily activities.

### Outcomes

All measurements in both the exercise group and control group were performed at the following time points, i.e., before TKA operation (pre-operation), before exercise (pre-exercise), at 12 weeks after the beginning of the circuit training program (mid-exercise), and after completion of the circuit training (post-exercise).

### Gait analysis

Gait analysis was performed by a three-dimensional, eight-camera motion capture system (VICON, Oxford Metrics, London, England) synchronized with two force platforms (OR6, AMTI, Watertown, Massachusetts) to record ground reaction force. Marker data were sampled at 100 Hz. Force platform data were sampled at 1000 Hz. Data collection starts with standing calibration to identify joint centers and create a segment impeded coordinate system. After calibration, subjects practiced walking until reaching a constant self-selected speed. The collected trials fell within 5% of the practiced speed with clear contact of only one foot on each force plate. Five walking trials were collected for each subject. While kinematics and kinetics of the segments are calculated, the whole body is modeled as a segment-linkage system consisting of the head, trunk, pelvis, bilateral upper arms, forearms, hands, thighs, shanks, and feet. Reflective markers attached on the segments were used to establish coordinate systems representing each segment. Data were processed utilizing the Nexus motion analysis system (VICON; Oxford Metrics Ltd. Ver.1.6.5), which was integrated with data recording software.

### Muscle strength

Lower extremity muscle strength (including extension and flexion of the hip and knee, dorsi, and plantar flexion of the ankle) was measured using the HUMAC NORM system (CSMi, Stoughton, MA) with the concentric/eccentric contraction mode at an angular velocity of 60° per second. All measurements were evaluated with the participant in a sitting position. Isokinetic tests were performed five times for each participant, and each test was separated by a rest period of 3 min. The participants received verbal encouragement during the exertion of peak torque. The muscle strength was presented as a peak torque which was normalized to body weight [15].

### KOOS assessment

Clinical knee scoring using the Chinese version KOOS scale was performed in the outpatient self-explanatory assessment [16]. The KOOS is a 42-item self-administered knee-specific questionnaire assessing pain (9 items), symptoms (7 items), activities of daily living (ADL, 17 items), function, sports and recreational activities (sports/rec, 5 items), and knee-related quality of life (QOL, 4 items) in five separate subscales. All items are scored 0 to 4; for each subscale, the scores are transformed to a 0 to 100 scales (0 representing extreme knee problems and 100 representing no knee problems) [17].

### Quality of life

Quality of life was assessed using the Short-Form Health Survey (SF-36, Taiwan version [18]). The SF-36 questionnaire is a multi-purpose and short-form health survey, which is commonly used to evaluate patients’ quality of life in clinical practice. A total of eight domains were evaluated in this questionnaire including physical functioning (PF), role limitation due to physical problems (RP), bodily pain (BP), general health (GH), vitality (VT), social functioning (SF), role limitation due to emotional problem (RE), and mental health (MH). Additionally, the eight health domains can be used to provide physical component summary (PCS) and mental component summary (MCS) scores.

### Statistical analysis

All data analysis was done using the Statistical Package for the Social Sciences, Windows version 17.0 (SPSS, Chicago, IL, USA). All continuous data are presented as the mean ± standard deviation. Generalized estimating equations (GEEs) [19] were used for determining the differences between the exercise and control groups across the time period. A *p* value of < 0.05 was considered statistically significant.

## Results

The demographic data, including age, height, and weight was similar in the exercise and control groups (Table 1). The mean age was 72 and 70 years old for the exercise and control groups, respectively.

**Table 1:** Demography of participants

	Exercise group (*n* = 16)	Control group (*n* = 18)
Age (years)	72.1 ± 6.7	69.6 ± 8.2
Height (cm)	152.6 ± 5.6	154.3 ± 6.4
Weight (kg)	63.7 ± 6.0	63.2 ± 11.9
BMI (kg/m^2^)	27.5 ± 3.3	26.5 ± 4.0

Data presented as mean ± SD

In gait analysis, it was noted both exercise and control group were shown similar in gait parameters at pre-operation and pre-exercise evaluation. Three months after beginning exercise interventions, the stride length for exercise and control group were 101.6 ± 3.4 and 85.0 ± 5.5 cm, respectively (*p* = 0.01). Similarly, step length and step velocity were greater in the exercise group than those in control group. (*p* < 0.05) (Table 2). Meanwhile, the excursion of knee range of motion was 48.6 ± 1.2 and 44.5 ± 1.6 degree for exercise and control group, respectively (*p* < 0.05). The differences in stride length and excursion of knee range of motion (ROM) lasted till the post-exercise evaluation (Table 2). When comparison was performed within the individual group in temporal fashion, it was shown that stride length, stride velocity, step length, and step velocity increased in mid-excise assessment in the exercise group, while no such difference was noted in control group. The control group demonstrated increases in these parameters at post-exercise assessment (*p* < 0.05). Circuit training improved gait pattern at an earlier time point compared with the control group.

**Table 2:** Comparison of gait kinematics between two groups of the TKA patients

	Pre-operation			Pre-exercise			Mid-exercise			Post-exercise		
	Exercise	Control	*p*	Exercise	Control	*p*	Exercise	Control	*p*	Exercise	Control	*p*
Stride time (sec)	1.2 ± 0.1	1.2 ± 0.1	.709	1.2 ± 0.1	1.2 ± 0.1	.716	1.1 ± 0.1	1.1 ± 0.1	.988	1.1 ± 0.1**^,^***	1.1 ± 0.1**	.270
Stride length (cm)	85.6 ± 5.7	82.0 ± 5.8	.663	92.0 ± 5.0	81.5 ± 4.9	.136	101.6 ± 3.4**^,^***	85.0 ± 5.5	.010*	100.0 ± 4.3**^,^***	85.1 ± 5.2	.027*
Stride velocity (cm/s)	72.6 ± 6.0	70.4 ± 6.7	.808	78.2 ± 5.9	69.8 ± 5.5	.295	90.4 ± 4.8**^,^***	76.1 ± 5.8	.060	91.0 ± 5.8**^,^***^b^	80.5 ± 5.7**^,^***	.198
Step time (sec)	0.6 ± 0.1	0.6 ± 0.1	.633	0.6 ± 0.1	0.6 ± 0.1	.938	0.6 ± 0.1**^,^***	0.6 ± 0.1	.959	0.6 ± 0.1**^,^***	0.5 ± 0.1**^,^***	.383
Step length (cm)	42.6 ± 2.9	39.3 ± 3.0	.436	45.7 ± 2.6	40.5 ± 2.7	.158	50.9 ± 1.9**^,^***	42.6 ± 2.8	.013*	50.8 ± 2.3**^,^***	44.8 ± 2.9**^,^***	.102
Step velocity (cm/s)	69.6 ± 5.8	68.9 ± 7.0	.937	78.0 ± 5.4	71.1 ± 6.1	.400	92.2 ± 5.1**^,^***	77.0 ± 5.7	.046*	93.5 ± 5.8**^,^***	80.5 ± 6.6**^,^***	.136
Excursion of active knee ROM (^o^)	34.9 ± 2.8	35.7 ± 2.7	.836	44.0 ± 1.9**^,^***	40.2 ± 1.5	.112	48.6 ± 1.2**^,^***	44.5 ± 1.6**^,^***	.045*	51.0 ± 1.4**^,^***	44.8 ± 2.6**^,^***	.037*

Data presented as mean ± SD

A significant difference (*p* < 0.05) between two groups is calculated by GEEs

*p < .05, difference between groups; **p < .05, difference with pre-operation; ***p < .05, difference with pre-exercise

In isokinetic muscle strength assessment, it was shown that the maximal knee extensor torque for the exercise group was 24.4 ± 5.2, 31.4 ± 3.3, 44.0 ± 4.8, and 51.0 ± 5.9 N-m/Kg for pre-operation, pre-exercise, mid-exercise, and post-exercise, respectively (*p* < 0.05). Similar increases were demonstrated in the control group for knee extensor (Table 3). Indeed, it was shown that maximal muscle strength in hip extensor, hip flexor, knee extensor and knee flexor, ankle plantar flexor, and dorsiflexor all increased after TKA surgery in a temporal fashion for both exercise and control group (*p* < 0.05). Meanwhile, it was shown that there were no differences between the exercise and control group at all time point (Table 3). Meanwhile, we measured the muscular strength of lower extremity since previous studies have shown that knee extensor strength is closely correlated to functional performance, especially after TKA [20, 21].

**Table 3:** Comparison of muscle strength between the two groups of the TKA patient

	Pre-operation			Pre-exercise			Mid-exercise			Post-exercise		
(Nm/kg)	Exercise	Control	*p*	Exercise	Control	*p*	Exercise	Control	*p*	Exercise	Control	*p*
HE	40.9 ± 5.5	44.2 ± 10.3	.733	56.0 ± 5.2**	58.1 ± 5.0	.774	66.9 ± 7.2**	74.1 ± 9.4**	.543	80.4 ± 10.4**^,^***	84.0 ± 7.0**^,^***	.773
HF	14.6 ± 2.5	16.7 ± 3.5	.615	17.8 ± 2.4	23.1 ± 2.2**	.109	26.0 ± 3.0**^,^***	27.2 ± 3.6**	.807	26.2 ± 3.6**^,^***	33.3 ± 2.6**^,^***	.114
KE	24.4 ± 5.2	32.5 ± 7.2	.363	31.4 ± 3.3	38.2 ± 6.4	.345	44.0 ± 4.8**^,^***	56.2 ± 8.4**^,^***	.207	51.0 ± 5.9**^,^***	61.5 ± 6.6**^,^***	.236
KF	26.1 ± 3.5	23.2 ± 4.3	.608	34.2 ± 2.4**	39.1 ± 5.9**	.444	48.6 ± 4.0**^,^***	52.0 ± 8.3**^,^***	.705	49.8 ± 3.7**^,^***	55.3 ± 5.6**^,^***	.413
PF	20.0 ± 2.6	20.8 ± 4.7	.888	24.6 ± 3.0	23.6 ± 3.1	.805	34.6 ± 4.0**^,^***	34.0 ± 6.6	.939	32.7 ± 2.9**^,^***	41.7 ± 5.4**^,^***	.145
DF	2.5 ± 0.6	3.3 ± 1.1	.568	3.7 ± 0.7**^,^***^b^	4.3 ± 1.5	.718	5.3 ± 0.8**^,^***	7.8 ± 1.8**^,^***	.204	6.6 ± 1.2**	7.6 ± 1.8**^,^***	.309

Data presented as mean ± SD

A significant difference (*p* < 0.05) between two groups is calculated by GEEs

*HE* hip extension, *HF* hip flexion, *KE* knee extension, *KF* knee flexion, *PF* plantarflexion, *DF* dorsiflexion

*p < .05, difference between groups; **p < .05, difference with pre-operation; ***p < .05, difference with pre-exercise

In post-exercise KOOS assessment, KOOS total score was 78.7 ± 3.2 and 66.4 ± 4.1 for exercise and control group, respectively (*p* = 0.018). In the subcategorical assessment, these differences were observed in pain and ADL (*p* < 0.05) (Table 4). When comparison of KOOS was performed within the individual group, the temporal improvement was observed in symptoms, pain, ADL, QOL, and total score for both exercise and control group (*p* < 0.05) (Table 4). Interestingly, the sports subcategory was shown significantly improved from 17.1 ± 5.4 at pre-operative assessment to 48.3 ± 7.8 at post-exercise assessment in the exercise group (*p* < 0.05), while no such difference was shown in the control group.

**Table 4:** Comparison of KOOS subscales between two groups of the TKA patients

	Pre-operation			Pre-exercise			Mid-exercise			Post-exercise		
	Exercise	Control	*p*	Exercise	Control	*p*	Exercise	Control	*p*	Exercise	Control	*p*
Symptom	45.9 ± 5.3	49.4 ± 4.8	.628	61.6 ± 3.1**	67.4 ± 3.8**	.240	72.9 ± 3.2**^,^***	71.0 ± 3.6**	.700	82.7 ± 3.2**^,^***	72.4 ± 4.5**	.059
Pain	46.8 ± 9.1	47.3 ± 6.4	.964	71.6 ± 4.5**	70.5 ± 4.8**	.866	76.3 ± 4.2**	77.8 ± 5.0**	.813	84.0 ± 2.5**^,^***	73.8 ± 4.5**	.048*
ADL	48.8 ± 7.5	48.5 ± 5.7	.980	72.6 ± 5.1**	70.8 ± 4.8**	.792	77.1 ± 4.2**	73.5 ± 5.0**	.570	84.5 ± 2.7**^,^***	71.2 ± 5.1**	.020*
Sport	17.1 ± 5.4	28.6 ± 6.7	.182	51.3 ± 8.6**	38.8 ± 6.1	.240	38.7 ± 4.9**	34.8 ± 5.9	.607	48.3 ± 7.8**	33.7 ± 5.4	.123
QOL	38.1 ± 6.0	40.2 ± 4.0	.775	53.9 ± 5.1**	48.3 ± 3.6	.367	63.5 ± 4.6**	61.7 ± 4.2**^,^***	.778	67.6 ± 4.3**^,^***	60.7 ± 4.7**^,^***	.277
Total score	43.3 ± 6.3	44.7 ± 4.9	.856	66.2 ± 4.1**	64.2 ± 3.9**	.715	70.4 ± 3.0**	68.3 ± 4.4**	.698	78.7 ± 3.2**^,^***	66.4 ± 4.1**	.018*

Data presented as mean ± SD

A significant difference (*p* < 0.05) between two groups is calculated by GEEs

*p < .05, difference between groups; **p < .05, difference with pre-operation; ***p < .05, difference with pre-exercise

In the SF-36 questionnaire which included physical and mental domains, it was shown a significant increase in the domains of GH, SF, and PCS at the mid-exercise point in the exercise group compared to the control group (*p* < 0.05) (Table 5). The circuit training efficiently improved the GH, SF, and PCS at the earlier time point. At the post-exercise assessment, only SF and BP were shown a significant increase in the exercise group compared with the control group (Table 5). When comparison was performed within the individual group in temporal fashion, it was shown that exercise improved all mental domains in mid-exercise assessment, and these improvements lasted to the post-exercise assessment, while no such improvement was observed in the control group. In the physical domain, it seemed both groups were shown improvement.

**Table 5:** Comparison of perceived health (SF-36) between two groups of the TKA patients

	Pre-operation			Pre-exercise			Mid-exercise			Post-exercise		
	Exercise	Control	*p*	Exercise	Control	*p*	Exercise	Control	*p*	Exercise	Control	*p*
PF	40.0 ± 4.8	35.2 ± 4.9	.487	49.1 ± 6.7	42.1 ± 6.3	.444	58.9 ± 4.5**	45.8 ± 5.5	.065	54.4 ± 6.5**	45.4 ± 6.6	.330
RP	31.7 ± 10.4	19.0 ± 9.2	.362	31.4 ± 10.4	50.2 ± 10.5**	.203	76.3 ± 9.**^,^***	48.0 ± 11.5**	.058	58.4 ± 12.9	51.4 ± 12.9**	.702
BP	36.0 ± 4.5	34.0 ± 4.6	.761	52.1 ± 5.0**	54.2 ± 5.0**	.763	64.1 ± 3.2**^,^***	57.7 ± 4.5**	.244	76.8 ± 3.7**^,^***	65.4 ± 4.1**	.038*
GH	49.9 ± 6.8	44.3 ± 5.3	.519	53.0 ± 5.3	50.5 ± 4.3	.715	62.5 ± 4.9***	46.1 ± 5.3	.022*	56.8 ± 5.8	49.3 ± 5.4	.340
VT	60.2 ± 4.5	55.5 ± 4.9	.477	65.5 ± 3.6	58.8 ± 4.4	.237	63.9 ± 3.9	63.9 ± 4.0	.986	71.2 ± 4.5**	64.1 ± 3.7	.220
SF	65.1 ± 7.4	61.4 ± 7.1	.715	66.5 ± 5.3	63.9 ± 4.6	.709	80.6 ± 3.6**^,^***	65.3 ± 4.7	.010*	82.8 ± 3.7**^,^***	68.6 ± 3.8	.008*
RE	43.8 ± 10.9	43.1 ± 10.7	.964	46.6 ± 11.2	65.5 ± 9.6	.138	75.5 ± 9.2**^,^***	62.7 ± 10.3	.352	76.1 ± 10.9**^,^***	64.3 ± 11.9	.465
MH	57.5 ± 4.3	56.2 ± 3.9	.823	64.9 ± 2.7	62.1 ± 2.9	.489	65.4 ± 3.6	65.5 ± 3.6	.995	68.5 ± 3.9**	63.0 ± 4.3	.343
PCS	35.5 ± 1.7	32.6 ± 1.7	.230	38.6 ± 2.1**	38.2 ± 1.4**	.874	45.6 ± 1.5**^,^***	38.5 ± 2.2**	.008*	43.9 ± 2.5**	40.4 ± 2.3**	.309
MCS	45.3 ± 2.4	44.8 ± 2.7	.900	46.9 ± 2.0	48.0 ± 1.8	.675	49.3 ± 2.1	48.4 ± 2.3	.766	51.8 ± 2.2**	48.6 ± 2.3	.318

Data presented as mean ± SD

A significant difference (*p* < 0.05) between two groups is calculated by GEEs

*p < .05, difference between groups; **p < .05, difference with pre-operation; ***p < .05, difference with pre-exercise

## Discussion

The most significant findings in the present study were that 24-week circuit training resulted in a decrease of pain and an increase of ADL and SF, along with an increase in stride length and excursion of knee ROM in gait analysis [8, 22, 23]. Second, the increases in all muscle strength were demonstrated in a temporal fashion in both exercise and control group [24]. Circuit training seemed not further increase the maximal muscle strength. Third, earlier recoveries in gait parameters, KOOS, and SF-36 at mid-exercise assessment for exercise group were well demonstrated. Although circuit training did not result in a further increase in maximal isokinetic muscle strength, it was postulated to enhance the muscle coordination which facilitated the performance of walking and daily function [25]. The improvement in pain after circuit training both in the KOOS and SF-36 further provided an important basis that knee can undergo unlimited swing and hence to increase the stride length [23].

In gait analysis, stride length and excursion of knee ROM in the exercise group were shown significant increases as compared to control group at the mid-exercise assessment, and these differences lasted to the post-exercise assessment (Table 2). It is well demonstrated that faster restoration of gait patterns in exercise group as compared to the control group. None of the subjects enrolled in the exercise group reported discomfort or injury, and none needed further management. This indicates that our circuit training intervention is safe for TKA patients and feasible to perform following the TKA surgery.

The function of quadriceps strength relied on maximal muscular strength and muscular coordination in TKA patients, which could be improved by post-operative progressive strengthening protocol [24, 26–29]. Quadriceps weakness is a hallmark characteristic of knee osteoarthritis [24, 27, 30, 31]. Post-operatively, quadriceps strength was not comparable to their age-matched counterparts in the mid- or long-term follow-up [29]. Muscle strength recovery in those aging patients underwent TKA followed a temporary pattern [24]. The current study displayed a simultaneous trend in pain and muscle function recovery. Actually, as the pain improved 3 months after TKA, the patient was able to load their knees and hence improve the lower extremity muscle force. As the pain improved more, it was shown that lower leg muscle strength has a corresponding further increase. We have previously demonstrated the progress of both cardiovascular fitness and muscle strength by circuit training and 12-week circuit training in healthy middle-aged women (aged 45–75) [12]. However, the circuit training in the current study did not result in a further increase in the maximal isokinetic muscle strength. Two possibilities existed. First, the intensity of resistant training in the current study could be inadequate and would not result in a further increase as compared to the daily activities [23]. Second, the maximal strength has plateaued. Further study was warranted to delineate the mechanism underlying the findings in the current study. On the other hand, the muscle coordination and performance were improved by circuit training as shown in gait parameters. This finding paralleled literature that modified gait efficacy scale was improved by exercise [23]. In this laboratory study, it was further shown a 15% increase in stride length, accompanied by 10% increase in excursion of knee ROM during walking. These results provided a solid basis to explain the positive effect of exercise on gait function. It was possible that the decrease in pain and a more coordinated muscle function improved the gait function.

Decreased exercise adherence usually endangered the effect of exercise intervention [10, 32]. The motives for exercise participation among patients with knee OA was suggested mainly as positive outcome expectation [10]. Other factors such as social interaction and enjoyment of exercise have been described as important facilitators of exercise behavior [22, 32]. The present study demonstrated the improvement in social function which could result in exercise adherence to 80%, which was comparable in OA patient without TKA [10, 22]. Our results supported that a more balanced exercise program in patients with TKA to achieve a better exercise adherence and consequent improvement in gait and function [11]. Furthermore, the present study provided a quantitative data regarding the exercise prescription with efficacy and efficiency, which could be practiced for months. These results could be the basis for developing home exercise regimens.

Several limitations in this study must be acknowledged. First, the small sample size (34 patients) in this study resulted in under power to show a statistical difference between two groups in head-to-head comparisons. However, comparisons in repeated measure provided a better statistical power. Comparisons of repeated measurements in the same patient demonstrated the effect of exercise in a time sequence. Second, the study is limited to the Asian experience of knee arthroplasty. Third, this was a short-term study; thus, we were unable to assess the effect post-operative circuit training in long-term functional outcomes. The resistance training, though might be submaximal, in combination with aerobic exercise and stretching exercise help faster recovery in gait, functional score, and social function. Fourth, this study is a retrospective study. The results of circuit training on TKAs should be further proved by a prospective randomized clinical trial design. Fifth, there was a heterogeneous effect among the patients in the exercise. The previous investigation has shown that the carrier with polymorphism of monocarboxylate transporter 1 gene (A1470T) exhibits a worse lactate transport and influences the performance with high-intensity circuit training [33]. The genetic variance may be responsible for the heterogeneity of circuit training effect on the individual patients. The further study focused on the genetic test before training intervention was suggested.

## Conclusions

Our post-operative circuit training intervention is safe and effective in improving and hastening the functional recovery after TKA surgery, even without enhancement of lower limb muscle strength. This circuit training intervention might be incorporated into the standard post-operative rehabilitation protocol for TKA patients. The ultimate influence of circuit training on TKA needs further long-term investigation.

## Abbreviations

ADL: Activities of daily living
BP: Bodily pain
GEE: Generalized estimating equations
GH: General health
KOOS: Knee injury and Osteoarthritis Outcome Score
MCS: Mental component summary
MH: Mental health
OA: Osteoarthritis
PCS: Physical component summary
PF: Physical functioning
RE: Role Limitation due to emotional problem
ROM: Range of motion
RP: Role Limitation due to physical problems
SF: Social functioning
SF-36: Short-Form Health Survey
VT: Vitality
